# Independence of long-term contextual memory and short-term perceptual hypotheses: Evidence from contextual cueing of interrupted search

**DOI:** 10.3758/s13414-016-1246-9

**Published:** 2016-12-05

**Authors:** Bernhard Schlagbauer, Maurice Mink, Hermann J. Müller, Thomas Geyer

**Affiliations:** 1 Department Psychologie, Lehrstuhl für Allgemeine und Experimentelle Psychologie, Ludwig-Maximilians-Universität München, Leopoldstraße 13, 80802 München, Germany; 2 Graduate School of Systemic Neurosciences, Ludwig-Maximilians-Universität München, Planegg-Martinsried, Germany; 3 School of Psychology, Birkbeck College, University of London, London, UK

**Keywords:** Contextual cueing, Perceptual implicit memory, Eye movements

## Abstract

Observers are able to resume an interrupted search trial faster relative to responding to a new, unseen display. This finding of rapid resumption is attributed to short-term perceptual hypotheses generated on the current look and confirmed upon subsequent looks at the same display. It has been suggested that the contents of perceptual hypotheses are similar to those of other forms of memory acquired long-term through repeated exposure to the same search displays over the course of several trials, that is, the memory supporting “contextual cueing.” In three experiments, we investigated the relationship between short-term perceptual hypotheses and long-term contextual memory. The results indicated that long-term, contextual memory of repeated displays neither affected the generation nor the confirmation of short-term perceptual hypotheses for these displays. Furthermore, the analysis of eye movements suggests that long-term memory provides an initial benefit in guiding attention to the target, whereas in subsequent looks guidance is entirely based on short-term perceptual hypotheses. Overall, the results reveal a picture of both long- and short-term memory contributing to reliable performance gains in interrupted search, while exerting their effects in an independent manner.

## Introduction

Searching for objects in the environment is one of the central capacities of the visual system and the underlying mechanisms are at the heart of many theories of visual processing (Treisman & Gelade, 1980; Wolfe, 1998). The role of memory, or past experience of visual information, in locating and identifying critical—target—items has long been debated, and it is now widely acknowledged that memory plays an important role in visual search (Enns & Lleras, 2008; Johnson, Woodman, Braun, & Luck, 2007; Kunar, Flusberg, Horowitz, & Wolfe, 2007; Peterson, Kramer, Wang, Irwin, & McCarley, 2001; Schankin & Schubö, 2010). However, relatively little is known about the relationship between different forms of memory and, with it, the interaction between different attention guidance signals. The current study is concerned with two such memory mechanisms that are of relevance for visual search: long-term contextual memory of target-distractor arrangements (Chun & Jiang, 1998) and short-term perceptual hypotheses about target and distractor locations (Lleras, Rensink, & Enns, 2005). Prior research showed that there are uncanny similarities in the configural representations underlying the two memory types (Jungé, Brady, & Chun 2009). Yet, no study has investigated the effects of contextual memory in a visual search task designed to study perceptual hypothesis testing—that is, interrupted search.

## Rapid resumption of interrupted search

Interrupted search is a modified visual search task designed especially for the investigation of memory-based visual search. In their original study, Lleras, Rensink, and Enns (2005) used a search array consisting of a target letter *T* within a set of distractor letter *L*s. The search display was presented in loops of on- and off-phases—the display was visible for 100 ms, followed by a blank display for 900 ms—until participants produced a response. Examination of the reaction time distribution resulting from interrupted search revealed that there were almost no (only approximately 4%) responses within 500 ms after the first presentation of the display. However, on the subsequent encounters, a substantial amount of responses did occur within 500 ms of the second, third, etc. presentation (30–50%). This effect has been referred to as “rapid resumption” of visual search and is attributed to some short-term memory of the previous search display. In more detail, the notion of rapid resumption builds upon a theory of re-entrant processing (Di Lollo, Enns, & Rensink, 2000) and assumes that visual search involves an iterative process of generating and testing perceptual hypotheses. Upon the presentation of a search array, a perceptual hypothesis relating to the target stimulus (e.g., its location) location is formed, which is then tested on the subsequent view of the display. If the hypothesis is confirmed, a response is elicited; if it is rejected, a new hypothesis is formed. Rapid resumption occurs when a perceptual hypothesis can be confirmed upon the reappearance of the display. In other words, if a correct hypothesis to the target is generated, it may be confirmed rapidly in the following look at the display, leading to expedited reaction times.

Follow-up experiments addressed the issue of the contents of perceptual hypotheses by introducing changes to the search displays across successive presentations; the rationale was that if the changed attributes are part of the perceptual hypothesis, this should reduce the rate of rapid resumption (Jungé et al., 2009; Lleras, Rensink, & Enns, 2007). It was found that changing the location of distractors nearby the target strongly affected rapid resumption, whereas the location of distractors more distant from the target had no detrimental effect. Furthermore, changes of the target orientation, i.e., of the response-relevant feature, had a negative effect on the rate of rapid resumption, but not changes of distractor orientation. Finally, rapid resumption was found to be modulated by task relevance, i.e., feature-based attention: repositioning distractors that shared the target’s color interfered with the effect, whereas repositioning distractors of a different color left the rate of rapid resumption unchanged. Jungé et al. (2009) concluded that perceptual hypotheses contain information about the location, but not the identity, of distractors in close proximity to the target item, as well as information about perceptual attributes of the target. Furthermore, Junge et al. surmised that rapid resumption is modulated by task relevance, in that only distractors of the target’s color (set) are incorporated in the perceptual hypothesis. This pattern of effects strongly resembles findings from another line of research on the memory-based guidance of visual search: contextual cueing.

## Contextual cueing of visual search

If a target object is consistently encountered within a stable spatial arrangement of task-irrelevant distractor objects, detecting the target becomes more efficient over time, relative to target detection in non-repeated arrangements. This contextual cueing effect is attributed to learnt target-distractor spatial associations stored in long-term memory, which come to guide the search (Chun, 2000; Chun & Jiang, 1998, 2003). Like in interrupted search, in typical investigations of contextual cueing, participants are required to find a target *T* amongst distractor *L*s, however, with the display being visible until a response is issued. In a block of trials, usually 12 spatially different displays are presented repeatedly, that is, their arrangement is held constant across the experiment; the other 12 are nonrepeated displays, consisting of random target-distractor arrangements. Note that target positions in non-repeated displays are kept constant to equate target position repetition effects across the two conditions; this way the difference between repeated and nonrepeated displays can be unequivocally attributed to contextual learning of distractor configurations (Chun & Jiang, 1998; Jiang, Swallow, & Rosenbaum, 2013). Numerous studies on the contents of contextual memory have revealed strong parallels to the findings of Jungé et al. (2009). For instance, Olson and Chun (2002) examined the spatial extent of contextual memory by repeating only half of the distractors in a given display, which was found to be sufficient to generate a contextual-cueing effect. Later, it was shown that essentially only the distractors in close proximity to the target give rise to contextual cueing (Brady & Chun, 2007). Regarding the identity of the distractors, it was shown that identity (i.e., orientation) changes of the target and distractors in repeated displays did not interfere with contextual cueing (Chun & Jiang, 1998). This suggests that what is stored in contextual memory are spatial target-distractor relations, not target or distractor identities. In addition to spatial (proximity) factors and featural resolution, the third finding of Jungé et al. (2009) regarding the effects of featural attention in perceptual hypothesis testing also has analogue in contextual cueing: searching for a target of a predefined color among two sets of distractors of either the same color as the target or a different color did yield a reliable contextual-cueing effect when only the arrangements of the target-colored distractors were repeated (Geyer, Shi, & Müller, 2010; Jiang & Chun, 2001; Jiang & Leung, 2005).

In summary, contextual memory and perceptual hypotheses exhibit striking similarities in terms of their spatial and featural properties, allowing for the possibility that the two mechanisms are linked by a common mnemonic representation.

## Rationale of the present study

In three experiments, we examined for a relationship between contextual memory and perceptual hypotheses. Each experiment used repeated and nonrepeated displays presented in an interrupted search task. First, in a normal visual search task, participants learned a set of (12) repeated displays. Subsequently, participants performed an interrupted search task with the same set of—now learnt—repeated displays, intermixed with random, nonrepeated displays. In Experiment 1a, the display was visible for 100 ms and interrupted by a blank screen for 900 ms (Lleras et al., 2005). Experiment 1b was a replication of Experiment 1a, except that the on-phase was prolonged to 500 ms. We manipulated the time available for processing the interrupted search displays, because this factor has been shown to critically influence both the contextual-cueing and the rapid resumption effects. Regarding the former, there is ample evidence that the cueing effect is relatively sluggish in that it takes processing time for the effect to “kick in” (Geyer, Zehetleitner, & Müller, 2010; Kunar, Flusberg, & Wolfe, 2008; Ogawa & Kumada, 2008): cueing effects are larger when observers are provided with additional time for processing the displays. Applied to interrupted search, this could mean that although perceptual hypotheses based on the current search display and contextual cues from long-term memory may be linked via a common mnemonic representation, contextual cueing would simply be too slow to contribute to this representation and aid interrupted search. Regarding perceptual hypotheses, Lleras, Rensink, and Enns (2007) found that the task-relevant target feature alone is sufficient for rapid resumption to occur. However, Jungé et al. (2009) argued that this finding might be attributable to the timing implemented in the interrupted search task; Lleras et al. presented the display for (on-phases of) only 100 ms, which might limit the resolution, that is, the spatial extent/range, of perceptual hypotheses. The same might be true for our Experiment 1a, allowing for only relatively narrow perceptual hypotheses that lack information about distractor locations and thus decreasing potential effects of long-term—distractor—memory on perceptual hypothesis formation in interrupted search. Given this, Experiment 1b introduced extended presentation times of 500 ms to examine potential influences of this factor.

In Experiment 2, we recorded participants’ eye movements to gain further insights into potential effects of contextual cueing on perceptual hypothesis formation. Eye-movement analyses in interrupted search had revealed the distance of the fovea to the target to be a strong predictor of rapid resumption (Van Zoest, Lleras, Kingstone, & Enns, 2007): comparing the distance to the target on two looks prior to observers’ overt responses showed that, in case of a rapid-resumption response, fixations were closer to the target already on the second-to-last look.

## Hypotheses

**Fig. 1:**
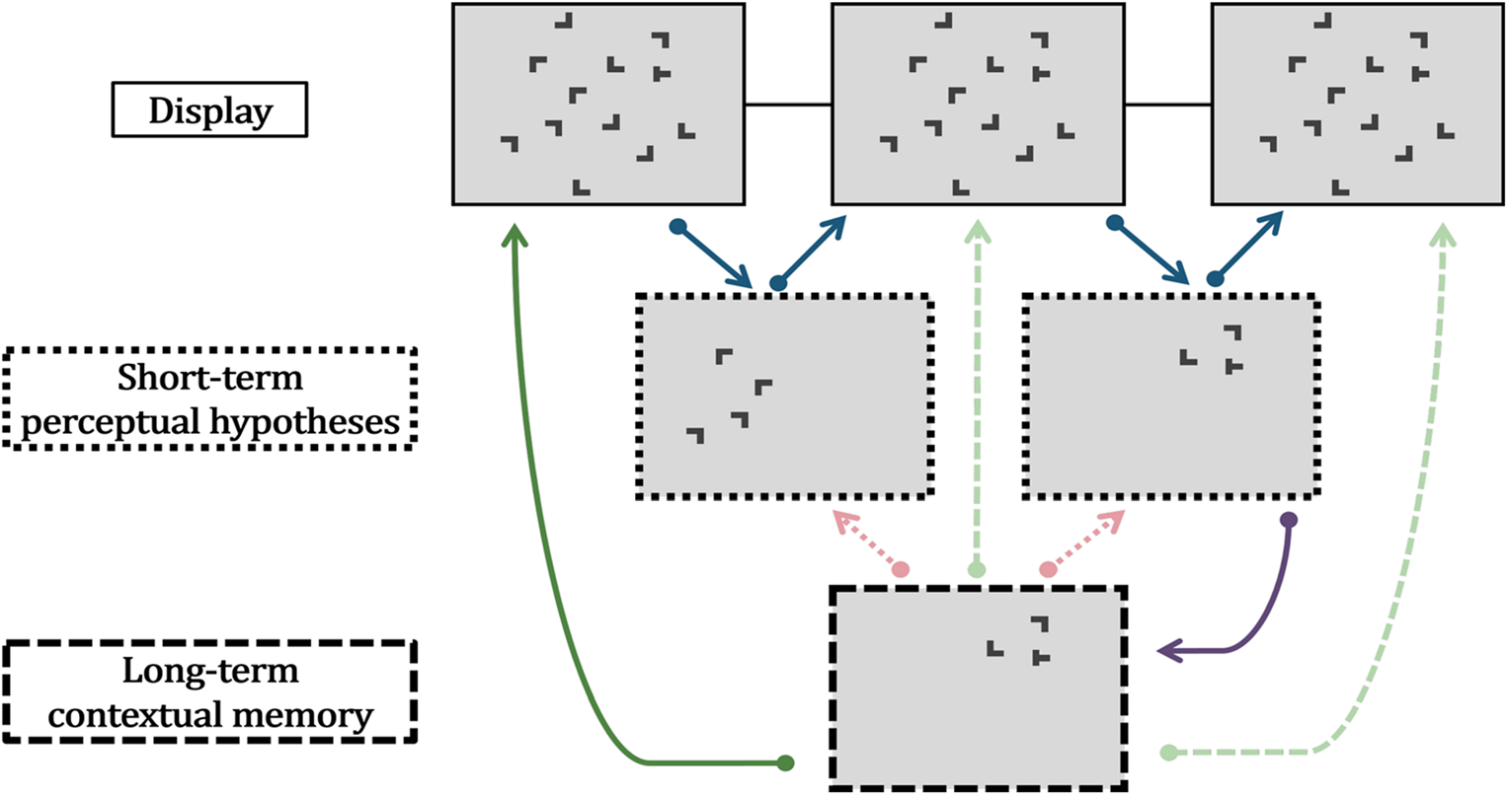
Schematic summary of the hypothesized relationship between short-term perceptual hypothesis and long-term contextual memory in the current study. In interrupted search, the displays are shown repeatedly for a short time, interrupted by blank displays for a longer time. Processing of the target and various distractor elements is influenced by short-term and long-term memory representations (middle and bottom panels, respectively). *Blue arrows* indicate the formation and test of perceptual hypotheses based on the current look (Lleras et al., 2005). The *purple arrow* indicates the build-up of long-term contextual memory, possibly based on the perceptual hypothesis (Jungé et al., 2009). *Red arrows* indicate possible influences of contextual memory on the formation or test of perceptual hypotheses (examined in the present study). *Green arrows* indicate possible influences of contextual memory on interrupted search, independent of short-term perceptual hypotheses (examined in the present study)

The presence of both types of perceptual memory—contextual memory and perceptual hypotheses—may result in either synergistic, interactive or independent, additive effects in interrupted search (Fig. 1).

### Synergistic effects

The high similarity of the memories underlying rapid resumption and contextual cueing, in terms of their spatial and featural characteristics (Jungé et al., 2009), may be taken to suggest that the two types of memory are integrated in a common, search-guiding representation. Conceivably, perceptual hypotheses, formed and held in short-term memory, integrate contextual cues retrieved from long-term memory (Fig. 1, red arrows) with information extracted from the currently viewed search display (Fig. 1, blue arrows). This may be an interactive process: information from the current search display would be required for retrieving context cues, which in turn might constrain the extraction of display information. In this way, contextual memory might expedite the *generation* of perceptual hypotheses, manifesting in an accelerated approach of fixations towards the target location and, thus, overall gains in search performance.

Alternatively, or in addition, the effects of contextual memory might become manifest at a later stage of the search process, namely: hypothesis confirmation. Contextual cueing also has been shown to facilitate processes of response selection (Kunar et al., 2007; Schankin & Schubö, 2010). If the target’s response-relevant feature is already incorporated in the perceptual hypothesis (Jungé et al., 2009; Lleras et al., 2007), it is possible that contextual cueing particularly expedites the mapping of a stimulus onto a response during perceptual hypothesis *confirmation*, resulting in a higher rate of rapid resumption or faster reaction times in the epoch of response.

### Independent effects

On the other hand, the relationship between perceptual hypotheses and contextual memory may be fairly loose; both mechanisms exert their effects in an independent fashion. For instance, due to the nature of the interrupted search task, perceptual hypotheses may receive dominant input from the current visual search display. Because the search display is available only for a short time, observers may focus their efforts on processing selected parts of the display. This may permit them to generate an already highly precise hypothesis about the target based on the current look, which cannot be further improved by guidance signals from long-term memory.

An alternative proposal is that perceptual hypotheses based on the current look occupy short-term memory resources, interfering with contextual cueing. This idea derives from recent findings that contextual cueing is reduced substantially when observers perform a demanding, secondary spatial working-memory task (Annac et al., 2013; Manginelli, Langer, & Pollmann, 2013). This is not to argue that the visual short-term memory underlying perceptual hypotheses is equivalent to working memory. However, there is evidence from both behavioral and neuroscientific investigations that visual short-term memory and working memory share functions and neural resources (Kristjánsson, Saevarsson, & Driver, 2013; Soto, Llewelyn, & Silvanto, 2012). The implications for interrupted search would be that the effects of contextual cueing on perceptual hypothesis formation and testing are weak, at best.

However, contextual cueing may nevertheless aid interrupted search by affecting parts of the search process that are unrelated to the formation and test of perceptual hypotheses (Fig. 1, green arrows). For example, in an oculomotor investigation of the cueing effect, Peterson and Kramer (2001) showed that the distance of the first fixation to the target and the angle of the first fixation from the target were smaller for repeated displays. Subsequent analysis showed that these effects were due to a higher proportion of initial fixations that were made directly to the target location. Peterson and Kramer took this to mean that contextual cueing is highly accurate in guiding attention to the target location (given successful recognition of repeated displays). Applied to interrupted search, this could mean that cueing modulates the starting point of the search process in that it biases attention towards certain displays parts, effectively shortening the interrupted search process. These performance gains would impact search early—before observers begin to search for the target in an iterative process of perceptual hypothesis generation and confirmation (Fig. 1, leftmost green arrow). Insofar, contextual cueing and the memory underlying perceptual hypothesis would exert their effects in an independent manner.

## Experiment 1a – Short viewing time

### Methods

#### Participants

**Fig. 2:**
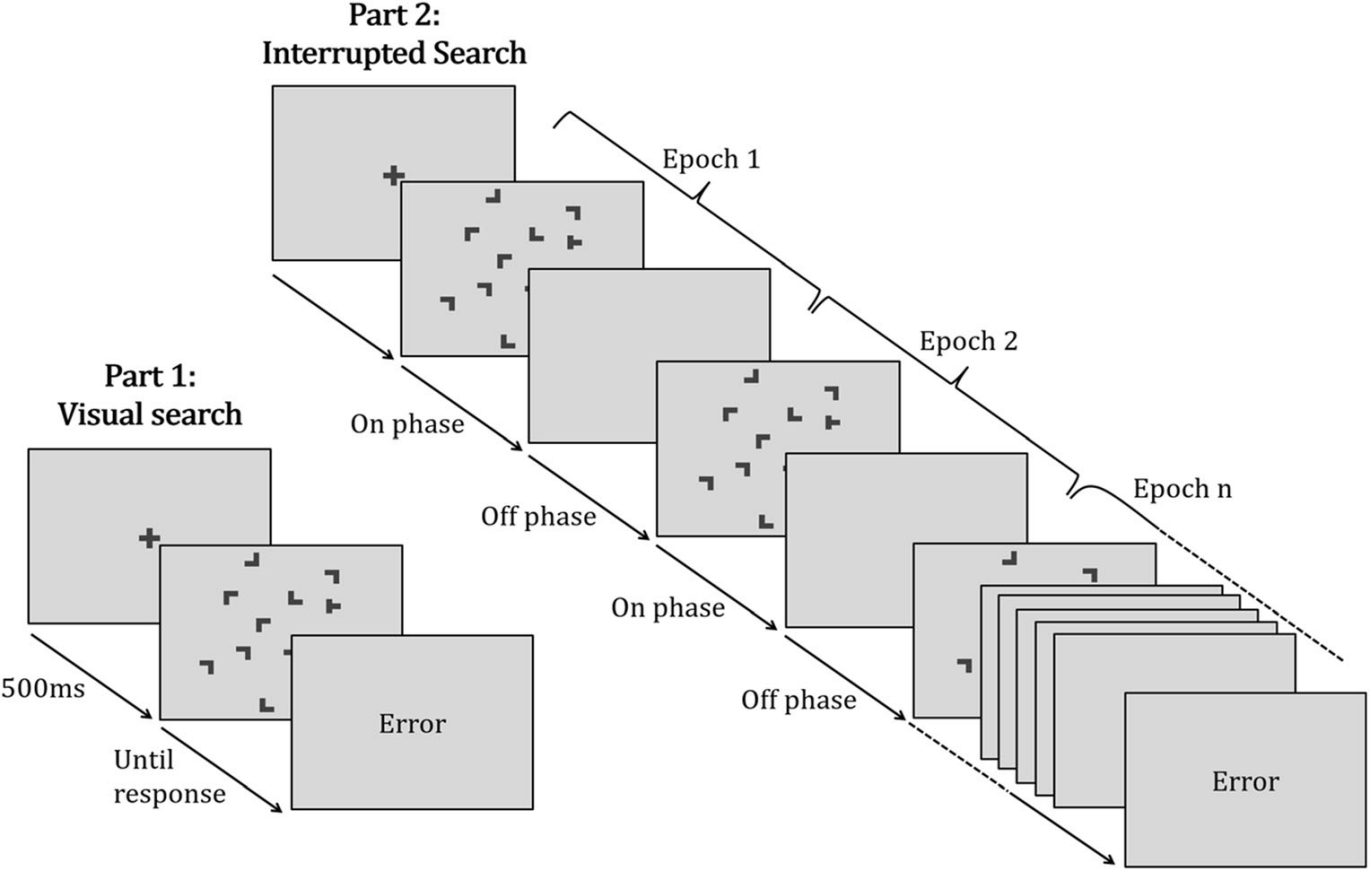
Sequence of events in a given trial of the visual search task (part 1) and interrupted search task (part 2). The procedure was identical for all experiments, except that part 2 differed with respect to the timing of the on- and off-phases (on-phase, Experiments 1a, 2: 100 ms; Experiment 1b: 500 ms; off-phase, Experiments 1a, 1b: 900 ms; Experiment 2: 1500 ms). In interrupted search, each display was presented repeatedly for a short period of time (on-phase) followed by a blank screen (off-phase), until a response was given

Twenty participants (5 males, 1 left-handed, mean age: 24.7 years) took part in Experiment 1a. All reported normal or corrected-to-normal vision. All participants provided written, informed consent before the experiment and received 8 € (~8.9 USD) or course credit for their participation. All participants received standardized written instructions for the two parts of the experiment (Fig. 2). Before the start of the experiment, participants performed two practice blocks: one with the visual search and one with the interrupted search task.

#### Apparatus and stimuli

The experimental routine was programmed in Matlab with the Psychtoolbox extension (Brainard, 1997; Pelli, 1997) and was run on a PC under the Windows XP operating system. Participants were seated in a dimly lit room in front of a 19-inch CRT monitor (display resolution 1024 x 768 pixels; refresh rate: 85 Hz) at a viewing distance of approximately 50 cm. All items in a given search array were dark grey (1.0 cd/m^2^), extended 0.5° of visual angle in width and height, and were presented against a light grey background (25.4 cd/m^2^). A given search array always consisted of 11 L-shaped distractors and one T-shaped target. Item positions were chosen pseudo-randomly such that display extension and item density were comparable across displays. Distractors were positioned on four (imaginary) concentric circles around the display center (radii: 2.32°, 4.64°, 6.96°, and 9.28°) with a minimum distance of 2.32° from each other. At least one item was positioned on each circle, and an equal number of items were positioned inside each quadrant of the display. The target was always located on the third ring from the center. There was a total of 24 possible target locations, 12 of which (3 in each quadrant) were used for repeated displays with constant distractor layout throughout the experiment. The other 12 target locations (3 in each quadrant) were used for nonrepeated displays with random distractor arrangements. The T-shaped target was oriented randomly either 90° to the left or 90° to the right; the L-shaped distractors were rotated either 0°, 90°, 180°, or 270°.

#### Task and procedure

The sequence of events in Experiment 1a (and Experiments 1b, 2) is depicted in Fig. 2. Participants first completed the visual search task, followed by the interrupted search task.

A visual search trial started with the presentation of a fixation cross (0.5° x 0.5°, 1.0 cd/m^2^) for 500 ms, followed by a blank interval of 200 ms before the search display was presented. Participants were instructed to respond as fast and accurately as possible to the orientation of the target letter T. If it was tilted to the right (left), they had to press the right (left) arrow button on a computer keyboard with their corresponding index fingers. Following an erroneous response, the word “Fehler” (German for error) appeared on screen for 1,500 ms, followed by a blank intertrial interval of 500 ms. A correct response immediately led to a blank screen, of 500-ms duration, before the start of the next trial. The visual search task consisted of 360 trials divided into 15 blocks of 24 trials each. Participants had the opportunity to take a short break between the blocks or continue directly with the next block. In each block, half of the trials contained repeated (old) displays with constant target-distractor arrangements. These 12 displays were generated randomly at the beginning of the experiment, but the arrangement was held constant across the entire experiment. The other 12 trials contained nonrepeated (new) displays with random distractor layouts (but constant target positions) generated anew on each trial. Trial order was randomized in each block. Target orientation was also a random variable.

An interrupted search trial started with the presentation of a fixation cross for 1,500 ms, followed by a blank interval of 200 ms. The search display was then presented for brief looks (on-phase: 100 ms), separated by longer waits (off-phase: 900 ms). Note that in the following, cycles of on- and off-phases are labelled as epochs. Participants were instructed to respond at any time during the trial to the orientation of the target letter T, while being as fast and as accurate as possible. If no response was given after 10 epochs—or 10 repeated presentations of the same display—the trial was terminated and counted as error trial. If an error occurred, the word “Fehler” (German for error) was presented for 1,500 ms followed by a blank intertrial interval of 500 ms. A correct response immediately triggered the blank interval of 500 ms. The interrupted search task also consisted of 360 trials divided into 15 blocks of 24 trials each. The 12 repeated displays used in part 1 also were used in part 2, making up half of the trials in each block. The other half were nonrepeated, random, displays with constant target positions (see above).

#### Data analysis

To acquire reliable estimates of contextual cueing, we collapsed five blocks into one *set* for analysis. Please note, that while most contextual cueing studies used the term *epoch* for aggregated blocks of trials (Chun & Jiang, 1998; Conci & von Mühlenen, 2009; Jiang & Chun, 2001; Peterson & Kramer, 2001), we tried to avoid confusion with terminology from the literature of interrupted search (Jungé et al. 2009; Lleras, Rensink, & Enns, 2005; Van Zoest, Lleras, Kingstone, & Enns, 2007) and used the term *epoch* solely to describe one cycle of on- and off-phase in an interrupted search trial.

To test our hypotheses conclusively, it was necessary to test for the absence of effects. Because nonsignificant results could only be interpreted as absence of evidence, we used Bayes factors, which also can be interpreted as evidence of absence if they are sufficiently small (Dienes, 2015; Rouder, Speckman, Sun, Morey, & Iverson, 2009). We computed Bayes factors using Bayesian linear models, equivalent to an ANOVA design. The Bayes factor of a given main effect or interaction is obtained by comparing a linear model including the effect of interest to a model which omits the effect (as implemented in the R package *BayesFactor* by Morey & Rouder, 2015). Participant number was always included as random effect. We used suggested default variance priors for linear models with a scale parameter of $\sqrt{2}/4$ (Rouder & Morey, 2012). A main effect or interaction was considered to be substantial if the Bayes factor was greater than 3. A Bayes factor less than 1/3 was considered as substantial evidence for the absence of a main effect or interaction (Wetzels et al., 2011). Bayes factor in between the thresholds indicate that the evidence for or against an effect is inconclusive.

In visual search, error trials were excluded from further analysis. Reaction times were analyzed with main terms for context (repeated vs. nonrepeated) and set (3 sets of 5 blocks each).

In interrupted search, error trials were excluded and we only analyzed trials with responses made within epochs one to six (see Lleras et al., 2005 for a similar procedure). Reaction times were analyzed with the same factors as in visual search (context: repeated, nonrepeated; set: 1, 2, 3) but separately for the two dependent variables *trial reaction times*—measured from the first presentation of the display until response—and *epoch reaction times*—measured from the last presentation of the display until response. Additionally, we examined the average epoch of response as a function of context and set. The rate of rapid resumption was compared between repeated and nonrepeated displays with a two-tailed paired Bayesian t-Test. We assumed a Cauchy distribution of the standardized effect sizes with the scale parameter r = $\sqrt{2}/2$ over the interval 0 to ∞, which has been suggested as a default prior in psychological research (Rouder et al., 2009). Responses with an epoch reaction time faster than the fastest individual reaction time in the first epoch but not slower than 500 ms were considered as rapid resumption responses (Lleras et al., 2005). Responses occurring later in an individual epoch were considered as normal search responses.

## Results

**Fig. 3:**
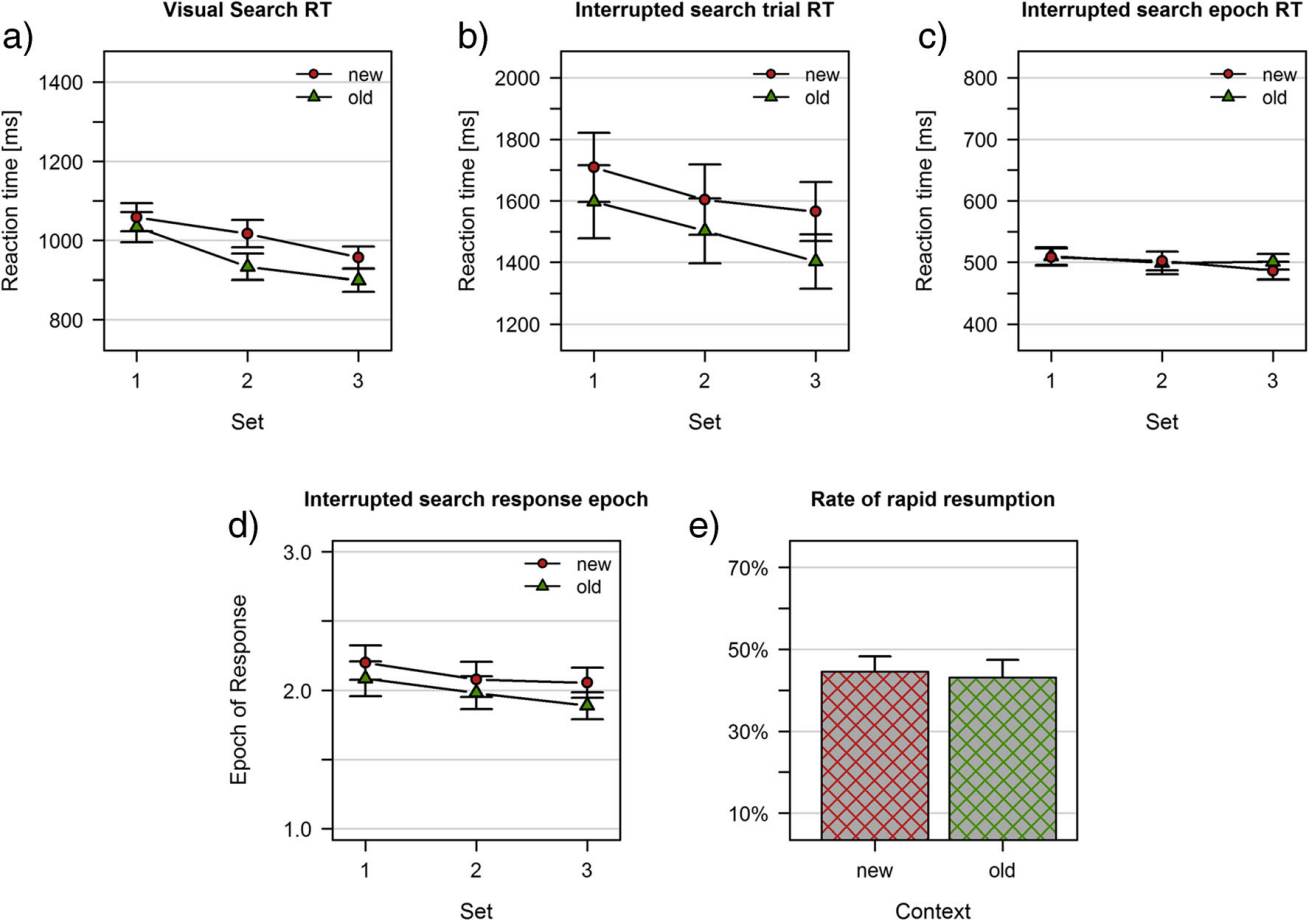
Results of Experiment 1a. **a** Visual search task; mean reaction times. **b**-**e** Interrupted search task: **b** Trial reaction times (reference is the first display onset). **c** Epoch reaction times (reference is the last display onset). **d** Average epoch of response. **e** Rate of rapid resumption trials. Data are shown separately for previously learned (old) and random baseline (new) displays

Data was analyzed using R (R Core Team, 2014), and Bayes factors were calculated using the package *BayesFactor* (Morey & Rouder, 2015). The results of Experiment 1a are depicted in Fig. 3.

### Visual search

The analysis of reaction times in visual search revealed substantial effects of context (*BF*_10_ = 27433) and set (*BF*_10_ = 3.144*10^11^) but no conclusive evidence for an interaction (*BF*_10_ = 1.09). The presence of a reliable contextual cueing effect suggests that repeated displays had been successfully learnt in the visual search task of Experiment 1a (Fig. 3a).

### Interrupted search

In interrupted search, trial reaction times indicate the total duration of a trial (i.e., time from the first onset of a display until the response). As depicted in Fig. 3b, there was substantial evidence for main effects of context (*BF*_10_ = 91.226) and set (*BF*_10_ = 84.503), in addition to substantial evidence for the absence of an interaction (*BF*_10_ = 0.171). Epoch reaction times indicate how fast participants responded after the last presentation of the display (i.e., time from the onset of the display in the epoch of response until the response; Fig. 3c). There was no conclusive evidence for a main effect of set (*BF*_10_ = 1.287) and substantial evidence for the absence of both an effect of context (*BF*_10_ = 0.264) and a context x set interaction (*BF*_10_ = 0.327). The analysis of the average epoch of response mirrored the pattern obtained from trial reaction times, with substantial evidence for main effects of context (*BF*_10_ = 99.179) and set (*BF*_10_ = 101.691), and substantial evidence for the absence of an interaction (*BF*_10_ = 0.180). Figure 3d illustrates the increased probability of repeated displays to yield responses in earlier epochs. The overall rapid resumption rate was at 43.88%, which was comparable between repeated and nonrepeated displays (old: 43.12%, new: 44.64%; *BF*_10_ = 0.241; Fig. 3e).

## Experiment 1b – Long viewing time

Twenty participants (8 females, all right-handed, mean age: 32.55 years) took part in Experiment 1b. Like in Experiment 1a, all participants provided written, informed consent, reported normal or corrected-to-normal vision, and received 8 € (~9 USD) or course credit for their participation.

The procedure of the experiment was identical to Experiment 1a, except for the timing in the interrupted search task: the on-phase was extended to 500 ms, so the display was visible for a longer period of time compared with Experiment 1a. The off-phase was kept at 900 ms. Because of the extension of the on-phase, an epoch in Experiment 1b lasted 1,400 ms.

## Results

### Visual search

**Fig. 4:**
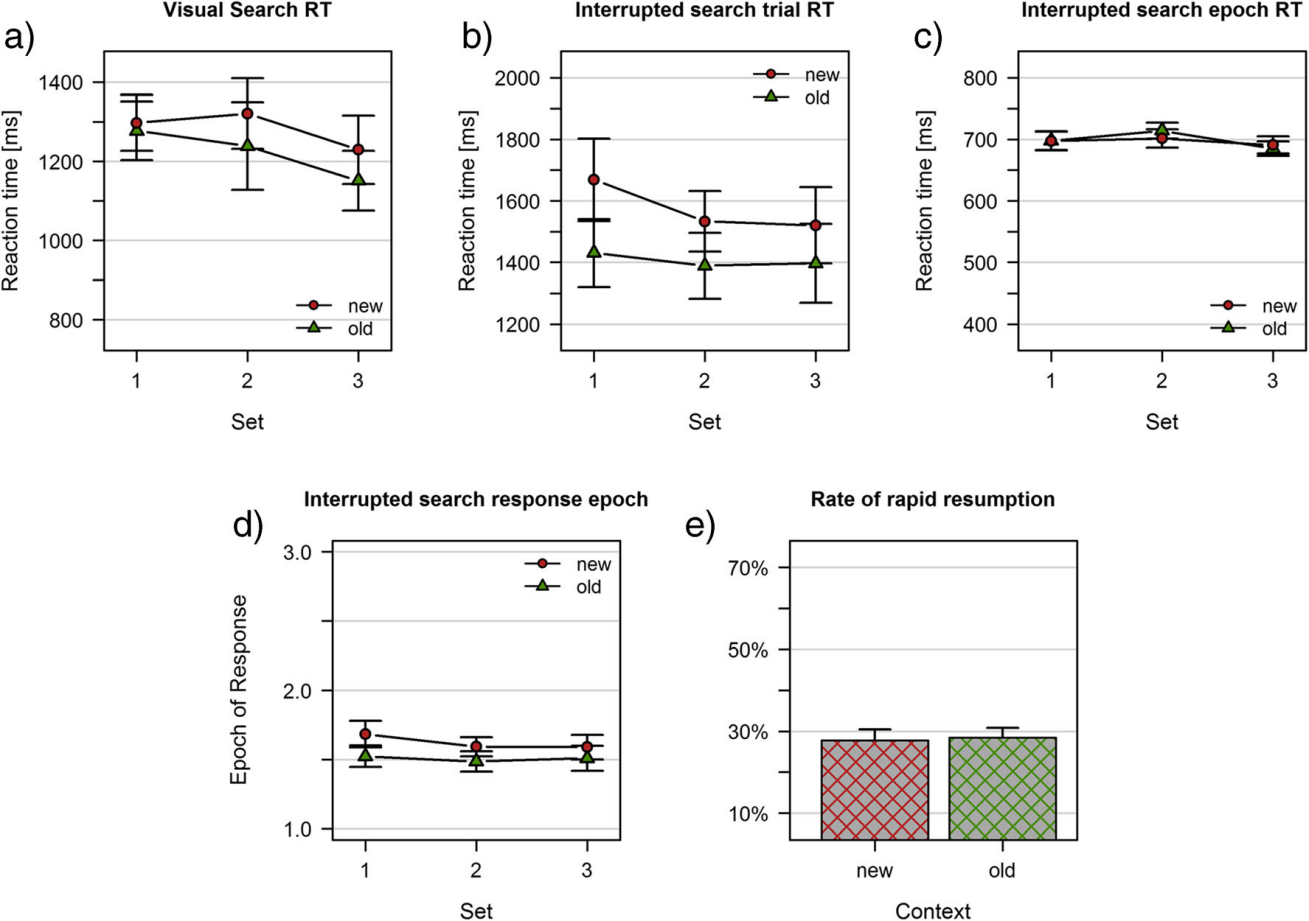
Results of Experiment 1b. **a** Visual search task; mean reaction times. **b**-**e** Interrupted search task: **b** Trial reaction times (reference is the first display onset). **c** Epoch reaction times (reference is the last display onset). **d** Average epoch of response. **e** Rate of rapid resumption trials. Data are shown separately for previously learned (old) and random baseline (new) displays

The analysis of reaction times in visual search revealed substantial evidence for main effects of context (*BF*_10_ = 11.424) and set (*BF*_10_ = 228.638) and for the absence of an interaction (*BF*_10_ = 0.291; Fig. 4a).

### Interrupted search

As depicted in Fig. 4b, trial reaction times in interrupted search yielded main effects of context (*BF*_10_ = 1.014*10^6^) and set (*BF*_10_ = 4.113); evidence for the absence of an interaction was inconclusive (*BF*_10_ = 0.483). The analysis of epoch reaction times revealed inconclusive evidence for an effect of set (*BF*_10_ = 1.287), but substantial evidence for the absence of a main effect of context (*BF*_10_ = 0.203) and an interaction (*BF*_10_ = 0.239; Fig. 4c). Analyzing the average epoch of response revealed a main effect of context (*BF*_10_ = 72231), whereas there was inconclusive evidence regarding a main effect of set (*BF*_10_ = 1.675) and an interaction (*BF*_10_ = 0.358). The overall rapid resumption rate was 28.06%, and there was substantial evidence for this rate being not different between old and new displays (old: 28.45%, new: 27.76%; *BF*_10_ = 0.190).

## Discussion

Experiments 1a and 1b combined interrupted search with contextual cueing by introducing previously learned spatial arrangements. In visual search (part 1), faster reaction times for repeated compared to non-repeated displays indicated that the stable target-distractor configurations were learned by participants and expedited visual search. This contextual cueing effect transferred to the interrupted search task (part 2), manifesting in overall shorter trial reaction times for repeated displays. However, analyses of epoch reaction times showed that the time after which observers executed a response in the last look at the search display was not affected by contextual memory. Instead, repeated displays elicited responses, on average, in earlier epochs. Furthermore, contextual memory did not affect the rate of rapid resumption. This pattern of results was identical for short and long viewing times (on-phases of 100 and 500 ms, respectively), making it unlikely that the nonfinding of effects of contextual cueing on rapid resumption was confounded by viewing times (see *Introduction*). An unexpected finding was that the overall rate of rapid resumption in Experiment 1b was reduced compared to Experiment 1a (1a: 43.88%, 1b: 28.06%, *BF*_10_ = 210), as was the general level of performance, indicated by overall slower reaction times in the first, visual search part of each experiment (1,252 ms in Experiment 1b vs. 983 ms in Experiment 1a: *BF*_10_ = 1.018*10^9^). However, because the pattern of results concerning repeated and non-repeated displays was the same in Experiments 1a and 1b, we interpret this as baseline shift due to differences in the collected samples rather than the experimental manipulation.

In summary, the findings suggest that the memory underlying rapid resumption does not benefit from long-term context memory of the same spatial layout, although overall performance was improved by long-term memory. This finding is at odds with the proposal that perceptual hypotheses and contextual memory have interactive, synergistic effects. Had perceptual hypothesis testing benefited from information stored in spatial long-term memory, one would have expected to observe either an increase in the rate of rapid resumption or faster reaction times in the epoch of response. Still, it might be the case that repeated displays had an advantageous effect on the generation of perceptual hypotheses, which could not be detected on the basis of observers’ manual responses. This is because trial reaction times are agnostic as to whether contextual cueing boosted the starting point of the search process—by effectively bringing attention closer to the target location already in the first look of the display, or whether context effects become manifest during the trial, effectively leading to more rapid generation of the correct perceptual hypothesis in subsequent looks. For this reason, in Experiment 2 we recorded eye movements (in addition to manual responses) to further explicate the processes during interrupted search and whether they are influenced by contextual long-term memory.

## Experiment 2 – Monitoring eye movements

### Methods

#### Participants

Thirty participants took part in Experiment 2. Four participants had to be excluded because of technical problems in recording eye movements (3 participants) or an excessively high error rate outside 2 standard deviations of the sample mean (1 participant; error rate: 29%). The remaining sample consisted of 26 participants (10 males, 1 left-handed, mean age: 24.23 years). Participants reported normal vision and did not wear any glasses or contact lenses. They provided written, informed consent before the experiment and received 12€ (~13.3USD) or course credit for their participation.

#### Apparatus and stimuli

The experimental setup was identical to Experiments 1a and 1b. Monocular eye movements of the dominant eye were recorded with an EyeLink 1000 system (SR Research, Canada), at a sampling rate of 1,000 Hz and a spatial resolution of 0.1° of visual angle. Head movements were minimized by a chin rest, placed 60 cm from the monitor. The experimenter operating the eye tracker during the experiment was seated outside the experimental booth.

#### Task and procedure

The task was identical to Experiment 1a, consisting of 15 blocks of visual search and 15 blocks of interrupted search. In interrupted search, displays were shown for an on-phase of 100 ms, with an interleaved off-phase of 1500 ms. Because Experiment 1b showed that an increase in viewing times did not further boost contextual cueing, one might speculate that retrieval from contextual memory might instead benefit from longer waits between display presentations. Therefore, the off-phase was prolonged to 1,500 ms in Experiment 2 (Experiments 1a and 1b: 900 ms).

#### Data analysis

The behavioral data was analyzed analogously to Experiments 1a and 1b. Eye movement data was analyzed by custom software implemented in Matlab using a data-driven and velocity-based detection algorithm described by Nyström and Holmqvist (2010). Statistical analysis of fixations was then carried out with R and Bayesian linear models, as described above. The crucial dependent variable was the distance of a fixation to the target stimulus, analyzed as a function of context (old vs. new), search type (normal search vs. rapid resumption), and epoch as temporal factor. Fixation distance to the target was analyzed in two ways: (a) response-locked, including in the analysis the epoch of response (look) and two epochs before the response (look-1 and look-2; see Van Zoest et al., 2007), and (b) stimulus-locked, including the first three epochs of a trial. An additional analysis was carried out in order to estimate across-look target approaching behavior by subjecting every trial to a linear regression. The fixation number (first, second, third fixation, etc.) was used to predict the distance of the fixation from the target. The resulting intercepts and slopes of every trial were then analyzed with a Bayesian linear model, with fixed effects of context and search type and participant number as random effect.

## Results

### Manual reaction time data

**Fig. 5:**
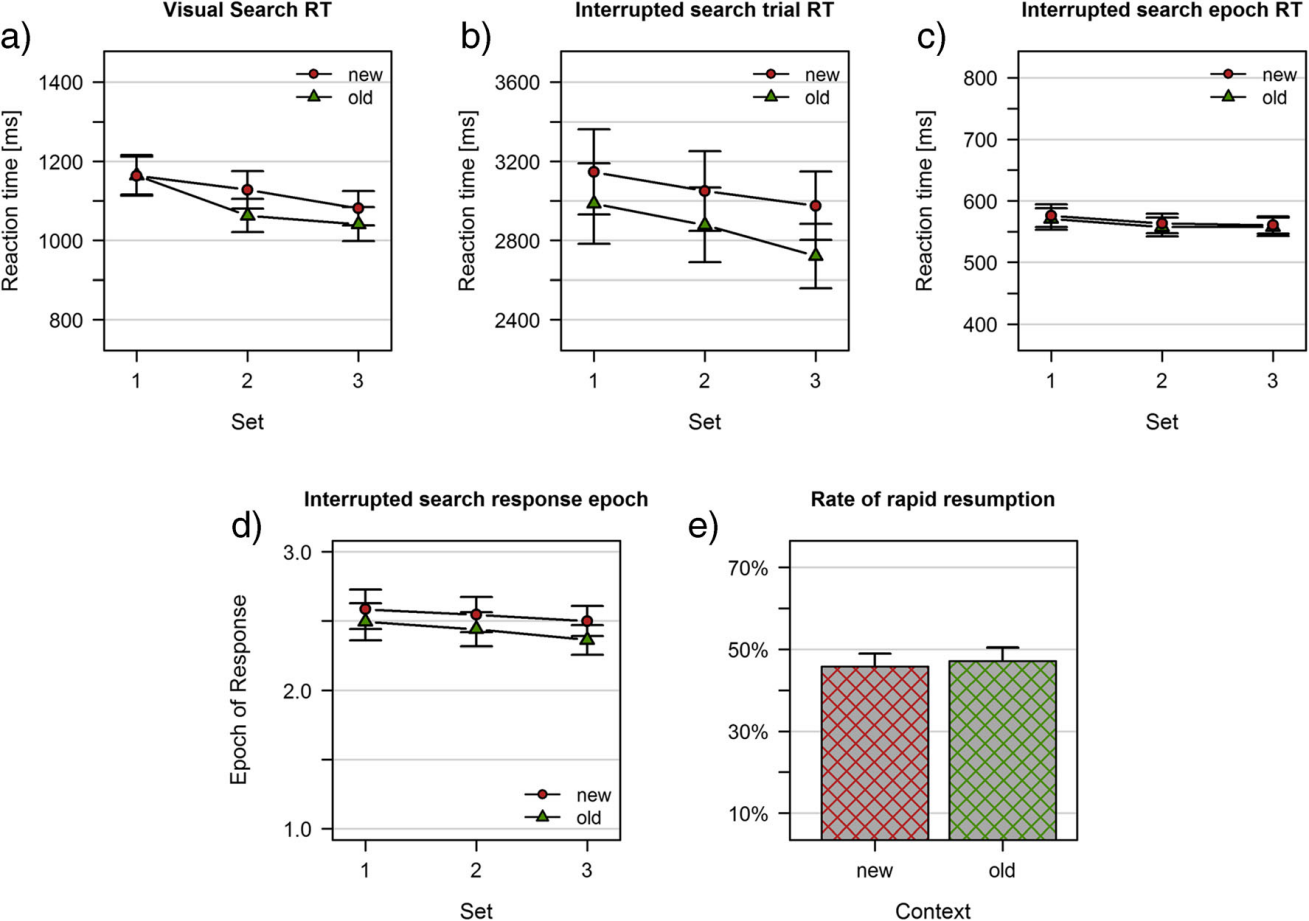
Results of Experiment 2. **a** Visual search task; mean reaction times. **b**-**e** Interrupted search task. **b** Trial reaction times (reference is the first display onset). **c** Epoch reaction times (reference is the last display onset). **d** Average epoch of response. **e** Rate of rapid resumption trials. Data are shown separately for previously learned (old) and random baseline (new) displays

Behavioral results are depicted in Fig. 5. Figure 5a shows reaction times in visual search, which yielded substantial main effects of context (*BF*_10_ = 45.306) and set (*BF*_10_ = 6.399*10^10^) and inconclusive evidence for an interaction (*BF*_10_ = 2.483). In interrupted search, analysis of the trial reaction times (Fig. 5b) revealed substantial main effects of context (*BF*_10_ = 73.289) and set (*BF*_10_ = 7.132) and an absence of an interaction (*BF*_10_ = 0.142). Figure 5c shows epoch reaction times produced substantial evidence for the absence of a main effect of context (*BF*_10_ =0.226) and of a context x set interaction (*BF*_10_ = 0.114), whereas evidence against a main effect of set was inconclusive (*BF*_10_ = 0.436). Regarding the average epoch of response (Fig. 5d), there was a substantial main effect of context (*BF*_10_ = 14.643) and substantial evidence for the absence of an interaction (*BF*_10_ = 0.124), whereas the evidence against a main effect of set was inconclusive (*BF*_10_ = 0.940). The overall rate of rapid resumption was at 46.47%. The test for a difference between old and new displays revealed substantial evidence for its absence (old: 47.21%, new: 45.80%; *BF*_10_ = 0.257; Fig. 5e).

### Eye movement data

**Fig. 6:**
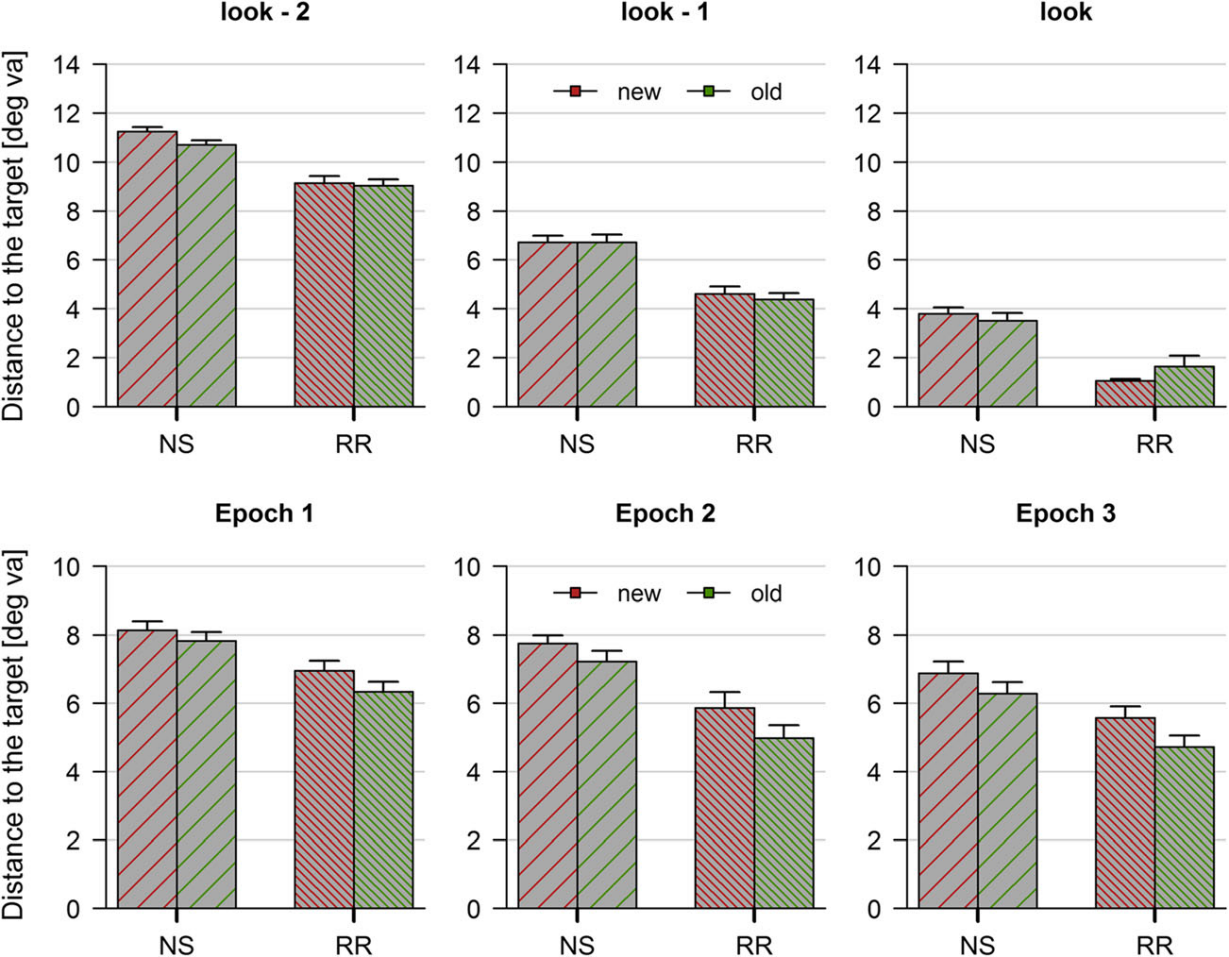
Results from Experiment 2. Distance in degrees of visual angle between the current fixation and the target, as a function of context (old vs. new) and search mode (normal search and rapid resumption). The *upper row* illustrates the response-locked analysis: fixations in the epoch of response and the two preceding epochs (look, look-1, and look-2). The *bottom row* illustrates the stimulus-locked analysis: fixations in the first 3 epochs of a given trial

Fixations in the interrupted search task were analyzed by calculating the distance of the first fixation in a given epoch, that is, the first fixation after the reappearance of the display, to the target stimulus (Fig. 6, top row). As expected, fixations were closer to the target on each consecutive look (main effect of look: *BF*_10_ = 6.697*10^124^). Furthermore, it was shown that rapid resumption responses were preceded by fixations closer to the target on three looks prior to the response (main effect of search type: *BF*_10_ = 9.316*10^33^). Regarding spatial context, there was no difference between old and new displays (absence of a main effect of context: *BF*_10_ = 0.152). None of the interactions were substantial, there was anecdotal evidence against an interaction of search type and context (*BF*_10_ = 0.372) and substantial evidence for the absence of all other interactions (*BF*’s_10_ < 0.298).

The stimulus-locked analysis, taking into account the first three epochs of each trial, revealed a different pattern of results. Again, search type (*BF*_10_ = 1.245*10^33^) and epoch (*BF*_10_ = 3.831*10^10^) yielded substantial main effects, indicating that the distance between the current fixation and the target was smaller for rapid resumption responses (main effect of search type) and that fixations came closer to the target in each consecutive epoch (main effect of epoch). However, there was substantial evidence for a main effect of context (*BF*_10_ = 590.190), indicating that fixations on trials with repeated displays were overall closer to the target location (Fig. 6, bottom row).

**Fig. 7:**
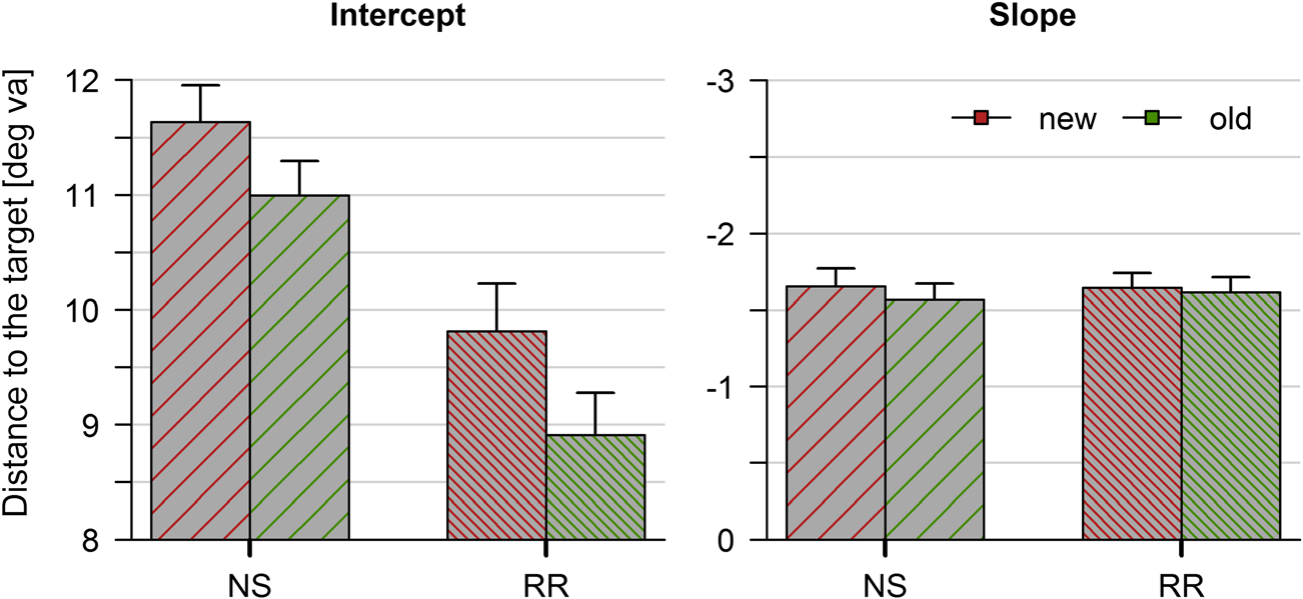
Comparison of regression parameters in Experiment 2. Slopes and intercepts were determined based on a linear regression of each trial with fixation number as predictor and distance to the target as criterion

To further illustrate this relationship, fixations were analyzed by means of a linear regression, using fixation number as predictor and distance to the target as criterion (Fig. 7). Subjecting the obtained intercepts to statistical analysis revealed that both search type (*BF*_10_ = 1.196*10^7^) and context (*BF*_10_ = 5.262) had substantial effects, yielding reliable decrements in the intercept of the fixation number x distance function. Importantly, there was substantial evidence for the absence of an interaction (*BF*_10_ = 0.304), suggesting that contextual cueing exerted a comparable influence on both normal and rapid resumption trials. The analysis of slopes, by contrast, revealed substantial evidence for the absence of both main effects and the interaction (*BF*s_10_ < 0.295).

## Discussion

Experiment 2 confirmed the results of Experiments 1a and 1b, demonstrating that contextual cueing and rapid resumption did neither interact in manual response nor oculomotor measures. As shown by Van Zoest et al. (2007), rapid resumption was evident in terms of closer fixations to the target already in the two epochs before the manual response; however, we could not replicate their finding that this was evident especially in the look prior to the response. Importantly, trials with repeated displays were statistically indistinguishable from trials with nonrepeated displays when analyzing target-fixation distances relative to observers’ manual responses. This indicates that the iterative process of generating and confirming perceptual hypotheses was not affected by long-term memory of the same spatial content. A contextual cueing effect was nevertheless expressed in the intercept of the function relating fixation number to the distance of the current fixation from the target location. This is indicative of an early, head-start effect of contextual memory in interrupted search, rather than an accelerated approach to the target during subsequent looks.

## General discussion

In three experiments, we investigated possible interactions between contextual long-term memory and short-term perceptual hypotheses, using the novel approach of presenting previously learned search displays in an interrupted search task. We observed both phenomena of contextual cueing and rapid resumption in isolation; however, we did not find evidence for joint effects. The behavioral results of all three experiments demonstrate that contextual memory was established during the visual search task and transferred to the interrupted search task. Yet, the rate of rapid resumption, as well as reaction times in the epoch of response were unaffected by contextual cueing. Instead, context effects became expressed through participants executing their responses in earlier epochs. Furthermore, the analysis of eye movements revealed that repeated and nonrepeated displays were indistinguishable from each other in terms of the speed with which the eyes approached the target stimulus. Instead, there was an initial effect on the distance of a given fixation from the target stimulus for learned versus novel target-distractor arrangements: interrupted search began with a shorter distance to the target item in repeated displays, while the search process itself was comparable between repeated and nonrepeated displays. Therefore, effects of long-term contextual memory on short-term perceptual hypotheses are ruled out by the current results (for this reason, the red arrows are blurred in Fig. 1), as well as influences of contextual memory on later stages of the interrupted search process (Fig. 1, blurred green arrows). The only reliable impact of contextual memory on interrupted search was an early effect on the initial processing of the display, before the formation or testing of perceptual hypotheses has commenced (Fig. 1, leftmost green arrow).

## The relationship of contextual memory and perceptual hypothesis-testing

Past studies provided converging evidence for strong parallels between rapid resumption and contextual cueing of visual search in terms of both spatial and featural properties (Jungé et al., 2009; Lleras et al., 2005). There are two possible processing stages where long-term and short-term memory of spatial context could have interacted in interrupted search, namely: the generation and confirmation of a perceptual hypothesis.

### Hypothesis generation

During interrupted search, contextual memory of repeated displays facilitated search performance, expressed by responses occurring in overall earlier epochs. This effect may indicate that contextual memory facilitates the generation of perceptual hypotheses, for instance, by providing some sort of guidance signal that aids in the process of forming a correct perceptual hypothesis. However, the analysis of eye movements revealed that the eyes approached the target stimulus at an identical speed in repeated and nonrepeated displays. If the generation of perceptual hypotheses—a process occurring iteratively over the course of the entire trial—was affected by contextual memory, one would have expected such effects to occur also within a trial. However, we observed an advantage for repeated contexts only when observers encountered the display for the first time. In the remainder of the trial, there was no evidence for contextual cueing to facilitate interrupted search. Therefore, we conclude that contextual memory influences only the very first generation of a perceptual hypothesis, providing a head-start for a search process which is then identical regardless of the presence or absence of long-term contextual cues. This is evidence against the idea of a synergistic relationship between contextual memory and perceptual hypotheses—as the effect of contextual cueing manifests only upon the first look, when there is no carryover of visual information from previous encounters of the search display.

### Hypothesis confirmation

Although the contents of contextual memory and perceptual hypotheses are very similar, we did not observe any modulation of rapid resumption for previously learned displays. In three experiments, Bayes factors provided substantial evidence for the absence of an increase (or decrease) of the rate of rapid resumption as a function of context. Rapid resumption is described as an effect of confirming a perceptual hypothesis generated during the previous presentations of the same display. While it has been suggested that contextual memory may be an instance of a perceptual hypothesis stored in long-term memory (Jungé et al., 2009), in the present study, this long-term memory representation did actually not influence the process of confirming an already established hypothesis, which also is supported by the finding of reaction times in the epoch of response being identical for repeated and nonrepeated displays. This may suggest that as soon as a strong search-guiding hypothesis is formed, long-term memory about this perceptual hypothesis is assigned a subordinate status. For instance, and as outlined in the Introduction, it is possible that bottom-up display cues and top-down context cues compete for limited resources in the memory underlying rapid resumption. If this memory was already filled with a perceptual hypothesis based on the current sensory input, less capacity would be left for hypotheses deriving from long-term memory. An alternative, although not mutually exclusive, view would be to assume that observers intentionally give priority to the processing of the current search display, for example, in an attempt to maximize information intake during the very limited viewing times. Future research may clarify why bottom-up display cues seem to play a major role in the generation and confirmation of perceptual hypotheses.

## Efficient vs. inefficient search

Our findings in Experiment 2 suggest that contextual memory influences the starting point of the interrupted search process and does not impact on the search process itself. Studies investigating eye movements in standard visual search usually report a reduction in the number of fixations owing to contextual cueing (Brockmole & Henderson, 2006; Manginelli & Pollmann, 2009; Peterson & Kramer, 2001), which goes along with a reduction in reaction times. Furthermore, Tseng and Li (2004) identified two stages of eye movement behavior in the contextual cueing of visual search: an initial phase of ineffective search and a phase of effective search, the latter characterized by the eyes systematically (monotonically) coming closer to the target item. They found that contextual memory of search displays led to a reduction of saccades only in inefficient search. Although an interrupted search task requires a different kind of search because the display is not constantly visible, the present pattern of results is strikingly similar to the “ineffective-effective” distinction in normal visual search. We observed that the average distance between a fixation and the target two looks prior to observers’ response were identical for repeated and non-repeated displays. On the other hand, contextual memory affected the intercept of the distance x fixation number function, with this parameter potentially reflecting the starting point for efficient search. Based on these findings, contextual cueing could be considered as mechanism that reduces the number of display items that would have to be inspected by attention to detect the target item. Therefore, our results support the findings of Tseng and Li (2004) in that long-term contextual memory has an early effect on the search trial, leaving the monotonic approach towards the target location unaffected. Applying this logic to the theory of reentrant processing (Di Lollo et al., 2000), context-based guidance of interrupted search could be described in the following way: The initial—inefficient—search is characterized by influences of long-term memory, setting up the starting point for the actual search process. Subsequently, this process continues by means of generating and confirming perceptual hypotheses, operating entirely on short-term visual memory of previous display exposures.

## Conclusions

The present investigation revealed that contextual cueing and perceptual hypotheses are rather independent memory phenomena, despite their high similarity in terms of both spatial and featural properties. Contextual cueing effects were observed in interrupted search; however, the effect became manifest only in a modulation of the starting point of the search process, while leaving the process of iteratively searching an interrupted display unaffected. The finding of an early cueing effect in interrupted search resembles findings from standard visual search showing a distinction between inefficient and efficient search within a single trial, with the effects of repeated contexts particularly contributing to a reduction of the inefficient phase. More specifically, conceiving of visual search as a two-stage process with first a parallel visual analysis followed by closer scrutiny of individual display items (Buetti, Cronin, Madison, Wang, & Lleras, 2016), contextual cueing would help to reduce the number of display items to be processed in the first, parallel stage.
